# miR-203 inhibits ovarian tumor metastasis by targeting BIRC5 and attenuating the TGFβ pathway

**DOI:** 10.1186/s13046-018-0906-0

**Published:** 2018-09-21

**Authors:** Baojin Wang, Xia Li, Guannan Zhao, Huan Yan, Peixin Dong, Hidemichi Watari, Michelle Sims, Wei Li, Lawrence M Pfeffer, Yuqi Guo, Junming Yue

**Affiliations:** 1 The Third Affiliated Hospital, Zhengzhou University, Zhengzhou, China; 2 Department of Pathology, the University of Tennessee Health Science Center, 19 S. Manassas St., Rm. 266, Memphis, TN 38163 USA; 3 Center for Cancer Research, University of Tennessee Health Science Center, Memphis, TN 38163 USA; 4 Henan Provincial People’s Hospital, Zhengzhou, China; 5 International Joint Laboratory for Gynecological Oncology Nanomedicine of Henan Province, Zhengzhou, China; 6 Department of Obstetrics and Gynecology, Hokkaido University School of Medicine, Hokkaido University, Sapporo, Japan; 7 Department of Pharmaceutical Sciences, the University of Tennessee Health Science Center, Memphis, TN 38163 USA

**Keywords:** miR-203, Ovarian cancer, Survivin, EMT, Tumor metastasis, Orthotopic ovarian cancer mouse model

## Abstract

**Background:**

We previously reported that miR-203 functions as a tumor suppressor in ovarian cancer cells by directly targeting transcription factor Snai2 and inhibiting epithelial to mesenchymal transition (EMT), whereas BIRC5/survivin promotes EMT. In this study, we tested our hypothesis that miR-203 inhibits ovarian tumor metastasis by suppressing EMT through targeting BIRC5, using an orthotopic ovarian cancer mouse model.

**Methods:**

We overexpressed miR-203 in ovarian cancer SKOV3 and OVCAR3 cells using a lentiviral vector and examined cell migration and invasion using transwell plates. The small molecule inhibitor, YM155, was used to inhibit survivin expression. miR-203-expressing and control SKOV3 cells were intrabursally injected into immunocompromised NSG female mice. Primary tumors in ovaries and metastatic tumors were collected to determine the expression of survivin and EMT markers using Western blot and immunostaining.

**Results:**

Overexpression of miR-203 inhibits EMT by targeting BIRC5 in ovarian cancer SKOV3 and OVCAR3 cells. miR-203 expression enhances the ability of the survivin inhibitor YM155 to reduce tumor cell migration and invasion in vitro*.* We further showed that miR-203 expression attenuated the TGFβ pathway in both SKOV3 and OVCAR3 cells. miR-203 expression also inhibited primary tumor growth in ovaries and metastatic tumors in multiple peritoneal organs including liver and spleen.

**Conclusion:**

miR-203 inhibits ovarian tumor metastasis by targeting BIRC5/survivin and attenuating the TGFβ pathway.

**Electronic supplementary material:**

The online version of this article (10.1186/s13046-018-0906-0) contains supplementary material, which is available to authorized users.

## Background

Ovarian cancer is one of the most lethal gynecological malignancies, with a five-year survival rate of only 30% due to intraperitoneal metastasis [1–3]. Most patients have no obvious symptoms and present with advanced to late stage disease that tumors already metastasized into multiple peritoneal organs at diagnosis [4]. The molecular mechanisms underlying the peritoneal metastasis is poorly understood. The epithelial to mesenchymal transition (EMT) is implicated in ovarian tumor metastasis and chemoresistance [5–10]. EMT is a process in which cancer cells lose their epithelial characteristics and acquire mesenchymal properties, thus promoting tumor cell invasion and metastasis. Thus, targeting EMT to inhibit tumor metastasis is an important focus for cancer therapy.

EMT is regulated by transcription factors including Snai1/2, ZEB1/2, and twist1 [11], and by multiple signaling pathways including TGFβ, AKT, ERK1/2, Notch, and WNT [12–15]. miRNAs function by negatively regulating the expression of their target genes at the post-transcriptional level through binding to the 3′ untranslated region of target genes. Previous studies showed that miRNAs regulate EMT in different type of cancer cells. miR-448 inhibits EMT by directly targeting E-cadherin repressor Zeb1/2 in breast cancer cells [16]. miR-302c inhibits EMT by targeting transcription factor AP1 in colorectal cancer [17]. miR-194 inhibits EMT by targeting Bmi1 in glioma [18]. In ovarian cancer, several miRNAs were also shown to regulate EMT by suppressing the expression of multiple target genes. miR-506, miR-382, miR-7, and miR-106 inhibit EMT by directly targeting Snai2, receptor tyrosine kinase orphan receptor 1 (ROR1), EGFR, and ZEB1/2 in ovarian cancer cells, respectively [19–22]. Taken together, these studies indicate that miRNAs are important regulators of EMT in different cancers.

We previously demonstrated that miR-203 is a tumor suppressor miRNA in ovarian cancer and that miR-203 expression inhibits tumor growth by targeting transcription factor Snai2 [23]. We also showed that BIRC5/survivin was highly expressed in ovarian cancer but not in the normal ovary tissues. BIRC5 expression promoted EMT, whereas knockout of BIRC5 inhibits EMT in ovarian cancer cells [24]. BIRC5 was reported to be a target gene of miR-203 in leukemia [25] and hepatocellular carcinoma (HCC) [26], which prompted us to hypothesize that miR-203 may regulate ovarian tumor metastasis by targeting BIRC5 in ovarian cancer cells.

In this study, we demonstrate that miR-203 expression inhibits EMT by targeting BIRC5 in ovarian cancer cells and enhances the efficacy of the survivin small molecule inhibitor YM155 on cell migration and invasion. miR-203 expression also inhibits ovarian tumor metastasis by targeting BIRC5 and attenuating the TGFβ pathway in an orthotopic ovarian cancer mouse model.

## Methods

### Cell culture

Ovarian cancer cell lines SKOV3 and OVCAR3 were purchased from ATCC and maintained in Dulbecco’s Modified Eagle Medium (DMEM) supplemented with 10% FBS (Hyclone; Logan, UT), 100 U/mL penicillin, and 100 μg/mL streptomycin (Invitrogen; Carlsbad, CA). miR-203-expressing and control SKOV3 and OVCAR3 stable cell lines were established by transducing both cell lines with lentiviral vector pEF1a-miR-203 vector, and miR-203 expression was detected by polyA tailing RT-PCR as described previously [23]. HEK293 FT cells were purchased from Invitrogen and cultured in DMEM supplemented with 10% FBS, 100 U/mL penicillin, 100 μg/mL streptomycin, and 1% glutamine.

### Transwell cell migration assay

Cell migration assays were performed using modified transwell chambers (BD Falcon™, San Jose, CA) inserted into 24-well culture plates. miR-203-expressing and control SKOV3 or OVCAR3 cells (3 × 10^4^) were suspended in 300 μL serum-free DMEM and added into the upper chamber. DMEM containing 10% FBS as the chemoattractant was added into the lower chamber of each well and incubated for 24 h. The medium and non-migrated cells in the upper chamber were removed, while the migrated cells on the lower side of the membranes were fixed with methanol and stained with crystal violet. Pictures were taken at 10X magnification, and cells from at least three different fields were counted.

### Wound healing cell migration assay

Both control and miR-203 expressing SKOV3 and OVCAR3 cells were treated with mitomycin for 4 h to inhibit cell proliferation and then cultured in serum-free medium for 24 h with or without YM155. Cell migration index was calculated as described as we published previously [27].

### Cell invasion assay

miR-203-expressing and control SKOV3 and OVCAR3 cells (5 × 10^5^) were seeded in serum-free DMEM onto inserts precoated with Matrigel (BD BioCoat™) using 24-well Tumor Invasion System (BD BioSciences, San Jose, CA). DMEM containing 10% FBS was added to the bottom chamber as the chemoattractant. The transwell inserts were fixed with methanol for 20 min and stained for 5 min with hematoxylin and eosin (H&E). Pictures were taken at 10X magnification and invaded cells were counted from at least three different fields.

### Immunofluorescent staining

To detect survivin and EMT markers in human ovarian cancer specimens or cell lines, ovarian tumor sections were antigen-retrieved by heating sections in sodium citrate buffer (10 mM sodium citrate, 0.05% Tween, pH 6.0) for 30 mins, while ovarian cancer cells were fixed in 4% paraformaldehyde for 30 mins. Sections or cells were incubated with blocking buffer (5% normal goat serum, 3% bovine serum albumin, and 0.1% Triton-X100 in PBS) for 1 h, and then incubated overnight with primary antibodies to survivin, PCNA, cytokeratin 7 and vimentin (1:200 dilution, Cell Signaling, Danvers, MA). After rinsing three times for 5 min with PBST, samples were incubated for 1 h at room temperature with Alexa 488- or 594-conjugated goat anti-rabbit (Invitrogen, Carlsbad, CA) antibodies. Cell nuclei were counterstained with DAPI (Vector Laboratories, Inc.; Burlingame, CA). Images were captured on a fluorescent microscope (Nikon, San Diego, CA).

### Western blot

Ovarian cancer cells were collected in RIPA buffer (Thermo Scientific; Rockford, IL) containing 1% Halt Proteinase Inhibitor Cocktail (Thermo Scientific). Equal amounts of protein (40 μg/lane) were loaded onto 10% SDS-PAGE gels and transferred onto nitrocellulose membranes. The membranes were blocked with 5% nonfat milk for 1 h and incubated with primary antibodies against survivin (1:1000, Cell signaling); GAPDH (1:5000, Sigma; St. Louis, MO); β-catenin, vimentin, Snai2 (1:1000, Cell Signaling), and cytokeratin 7 (1:1000, Abcam).

### Orthotopic ovarian cancer mouse model

All animal experiments were performed in accordance with a protocol approved by the Institutional Animal Care and Use Committee at the University of Tennessee Health Science Center. Only immunocompromised NOD.Cg-Prkdcscid Il2rgtm1Wjl/SzJ (NSG) mice (Jackson Laboratory) were used in this study. To generate an orthotopic ovarian cancer mouse model, miR-203-expressing and control stable SKOV3 cells were labelled with luciferase by transduction with the lentiviral vector pEF1a-Luc2. 1 × 10^5^ cells were intrabursally injected into five-week-old females by performing surgery under a dissecting microscopy. Tumor growth and dissemination in NSG mice were subjected to live animal imaging weekly to quantify bioluminescence, immediately after intraperitoneal injection with D-luciferin. Mice were sacrificed at 2-months following cell injection, and tumors were collected for histology, immunofluorescent staining, and Western blot to determine survivin and EMT marker expression.

### Statistical analysis

Significant differences were determined from at least two independent experiments performed in triplicate by Student’s *t-*test and data were presented as mean ± SD. *P* < 0.05 was considered significant.

## Results

### miR-203 inhibits EMT by targeting survivin in ovarian cancer cells

**Fig. 1:**
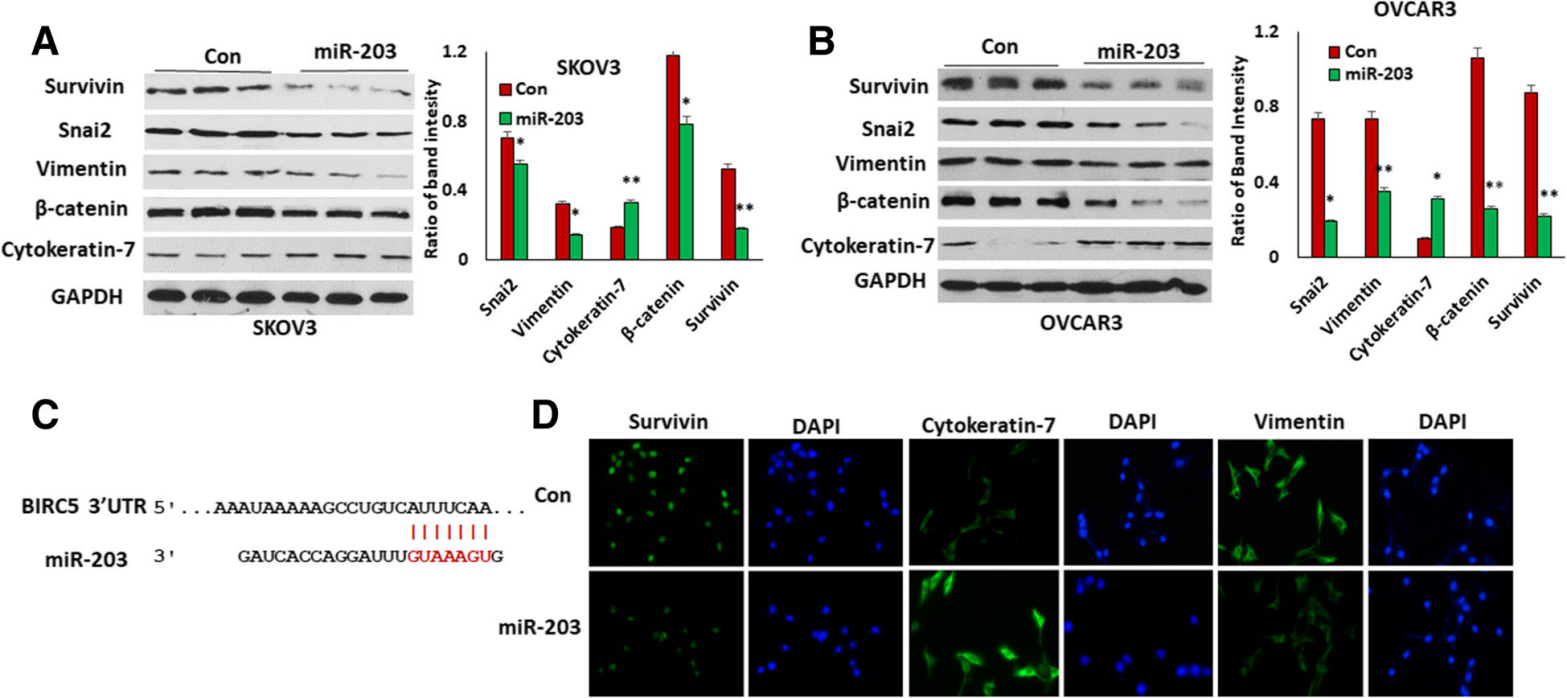
miR-203 inhibits EMT by targeting BIRC5 in ovarian cancer cells. **a**, **b**. Western blot analysis of EMT markers in miR-203-expressing SKOV3 (**a**) and OVCAR3 cells (**b**). **c**, Binding site of miR-203 in BIRC5 mRNA. **d**. Immunofluorescent staining of survivin and EMT markers in miR-203-expressing SKOV3 cells and controls

In ovarian cancer miR-203 expression is significantly reduced, while BIRC5 is highly expressed, as we published previously [23, 24]. miR-203 is inversely correlated with survivin in ovarian cancer [23]. BIRC5 has been shown to be a miR-203 target in leukemia and hepatocellular carcinoma [25, 26]. To examine whether BIRC5 is a target gene of miR-203 in ovarian cancer cells, we established miR-203-expressing SKOV3 and OVCAR3 stable cell lines using lentiviral vector as described previously [23]. BIRC5 and EMT marker gene expression was examined in both miR-203-expressing and control SKOV3 and OVCAR3 cells using Western blot. As shown in Figs. 1a and b, survivin was significantly downregulated in miR-203-expressing SKOV3 and OVCAR3 cells compared to control cells. Mesenchymal markers including Snai2, vimentin, and β-catenin were also downregulated, while the epithelial marker cytokeratin-7 was upregulated in miR-203-expressing SKOV3 and OVCAR3 cells. The miR-203 binding site in the 3’ UTR of BIRC5 is shown in Fig. 1c. We further validated expression of survivin and EMT markers, including cytokeratin-7 and vimentin, in miR-203-expressing and control SKOV3 cells using immunofluorescent staining. Survivin expression was stained in cell nuclei while vimentin was stained in cell membranes. Both survivin and vimentin expression were downregulated, whereas cytokeratin-7 expression (stained in cell membranes) was upregulated in miR-203-expressing SKOV3 cells compared to controls (Fig. 1d). These results showed that BIRC5 is a target gene of miR-203, and that miR-203 expression inhibits EMT in ovarian cancer cells by targeting BIRC5 in ovarian cancer cells.

### miR-203 expression augments the efficacy of the survivin inhibitor YM155 in inhibiting migration and invasion

**Fig. 2:**
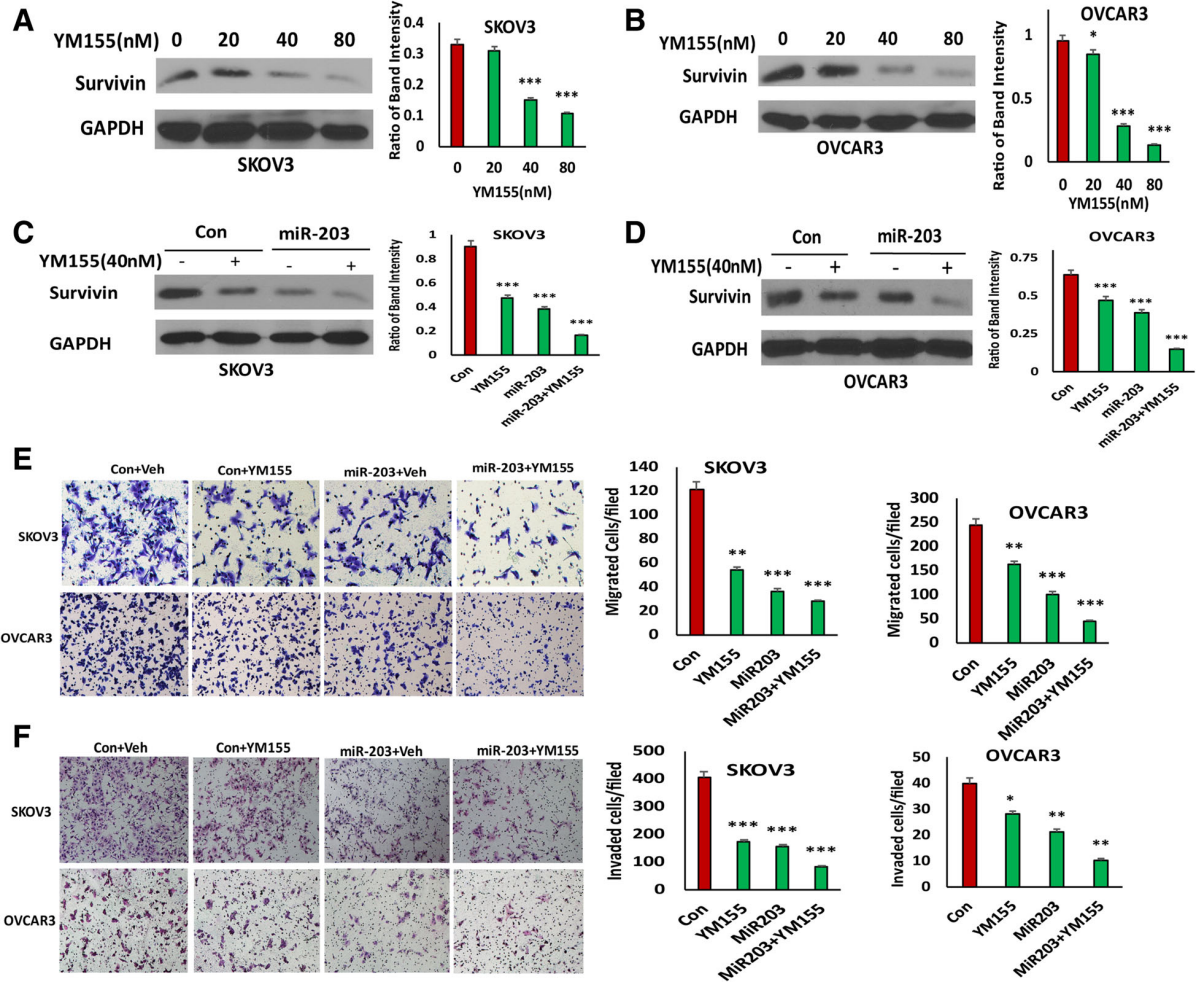
miR-203 expression augments efficacy of survivin inhibitor YM155 in cell migration and invasion. **a**, **b**. Western blot analysis of survivin in SKOV3 (**a**) and OVCAR3 (**b**) cells treated with different doses of YM155. **c**, **d**. Western blot analysis of survivin in miR-203-expressing and control SKOV3 and OVCAR3 cells. **e.** Cell migration of miR-203-expressing and control SKOV3 and OVCAR3 cells following 40 nM YM155 treatment (***p < 0.001). **f**. Cell invasion of miR-203-expressing and control SKOV3 and OVCAR3 cells following 40 nM YM155 treatment (***p < 0.001)

We previously showed that BIRC5 expression was upregulated in ovarian cancer compared to controls, and inhibition of BIRC5 expression using small molecule inhibitor of survivin YM155 leads to reduced cell migration and invasion [24]. Since BIRC5 is a target gene of miR-203, we hypothesized that miR-203 expression may enhance the efficacy of YM155 to inhibit cell migration and invasion. We first examined the effect of different doses of YM155 on survivin expression, and found that survivin was significantly reduced by YM155 at 40 or 80 nM in both SKOV3 and OVCAR3 cells, as shown by Western blot (Figs. 2a and b). To test whether miR-203 expression affects the efficacy of YM155, we treated miR-203-expressing and control SKOV3 and OVCAR3 cells with 40 nM YM155. miR-203 significantly reduced survivin expression, and addition of YM155 further reduced survivin expression in both cell lines as shown in Figs. 2c and d.

To further test whether miR-203 expression affects the ability of YM155 to inhibit cell migration and invasion, we performed cell migration and invasion assays with or without 40 nM YM155 in miR-203-expressing and control SKOV3 and OVCAR3 cells. miR-203 expression significantly enhanced the ability of YM155 to inhibit cell migration (Fig. 2e) and invasion (Fig. 2f) in both SKOV3 and OVCAR3 cells. In addition, we performed wound healing assay to validate our migration data to exclude the effect of cell proliferation following Mitomycin treatment for 4 h. Consistently, we obtained the similar results in both control and miR-203 expressing SKOV3 and OVCAR3 cells with or without YM155 (Additional file 1: Figure S1A, B) with that from transwell cell migration assay. Our data indicate that miR-203 enhanced the activity of the survivin inhibitor YM155 to inhibit ovarian cell migration and invasion.

### miR-203 expression attenuates the TGFβ pathway through targeting BIRC5

**Fig. 3:**
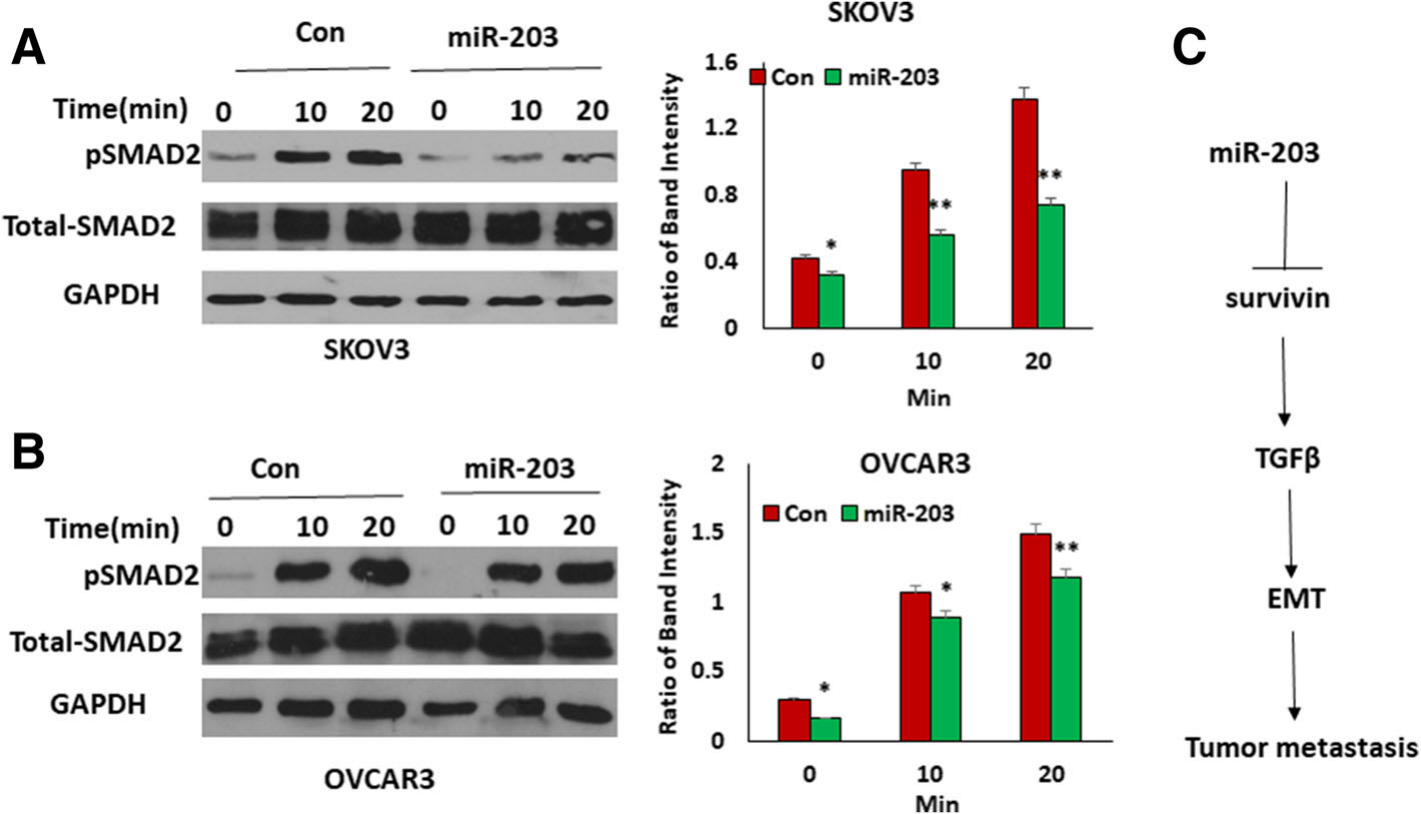
miR-203 expression attenuates the TGFβ pathway. **a**, **b**. Western blot analysis of phospho- and total SMAD2 in miR-203-expressing and control SKOV3 (**a**) and OVCAR3 (**b**) cells. **c**. A schematic diagram of miR-203 in attenuating the TGFβ pathway and inhibiting tumor metastasis

We showed previously that TGFβ promotes BIRC5 expression in ovarian cancer cells, and that knockout of BIRC5 or inhibition of BIRC5 with YM155 attenuated the TGFβ pathway [24]. Since BIRC5 is a target gene of miR-203, we hypothesized that miR-203 expression may attenuate the TGFβ pathway by targeting BIRC5. Therefore, we examined the TGFβ pathway in both miR-203-expressing and control SKOV3 and OVCAR3 cells by detecting phospho-SMAD2 using Western blot. As expected, miR-203 expression significantly attenuated phospho-SMAD2, but did not affect the total SMAD2 in both SKOV3 and OVCAR3 cell lines (Figs. 3a and b). These results suggested that miR-203 expression attenuates the TGFβ pathway by downregulating survivin, thus inhibiting EMT and tumor metastasis (Fig. 3c).

### miR-203 inhibits primary tumor growth and metastasis in an orthotopic ovarian cancer mouse model

**Fig. 4:**
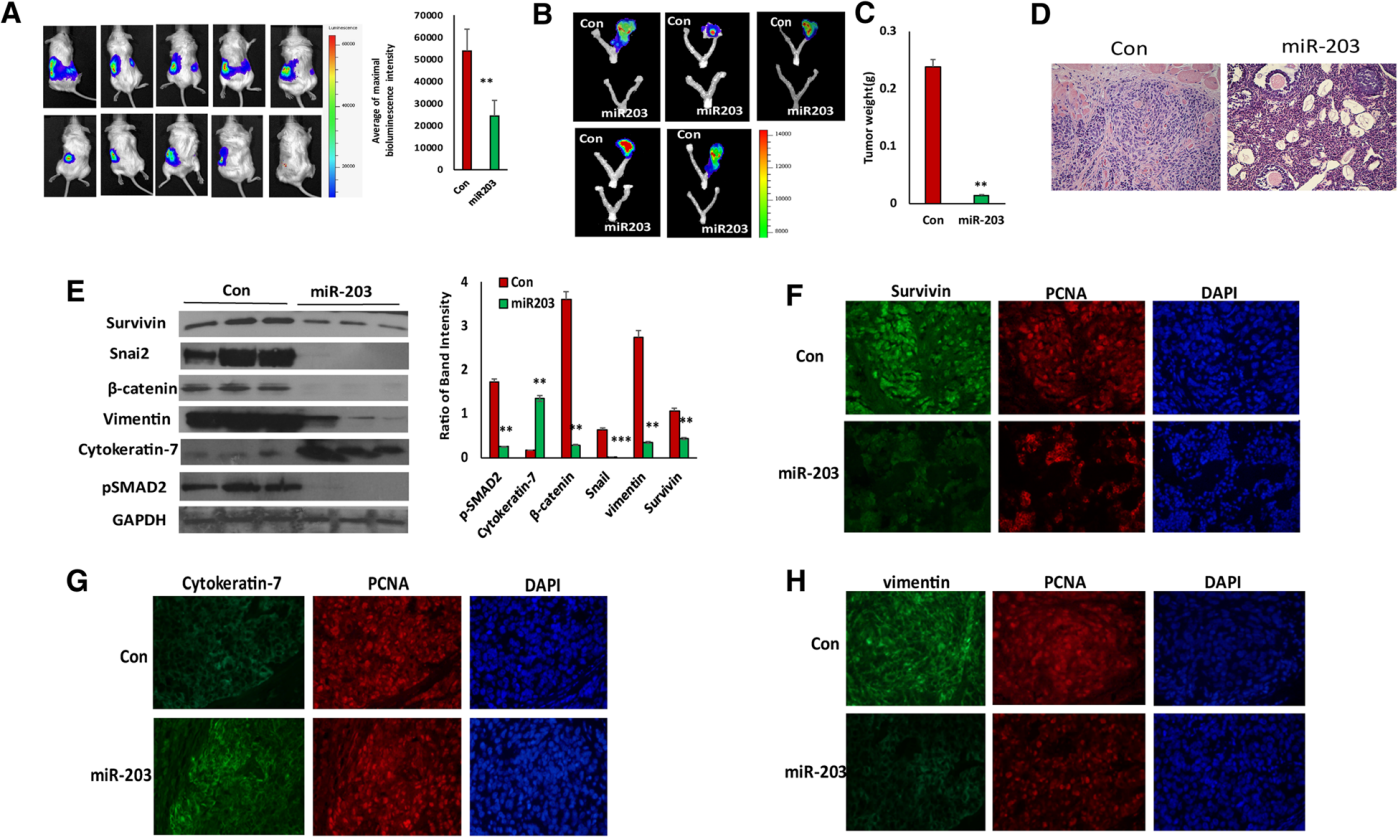
miR-203 expression inhibits primary tumor growth in ovaries. **a**, Bioimaging of mice at one month following intrabursal injection of miR-203 expressing and control SKOV3 cells. **b** Primary tumor in ovaries were imaged at two months following intrabursal injection (*n* = 5, **p < 0.01). **c**. Tumor weight in primary ovaries in mice intrabursally injected with miR-203-expressing and control SKOV3 cells (**p < 0.01). **d**. H&E stained sections of primary tumor in ovaries. **e**. Western blot analysis of survivin, EMT markers, and phospho- and total SMAD2 from tumors in ovaries (*n* = 3, **p < 0.01; ***p < 0.001). **f**, **g**, **h**. Immunofluorescent staining of survivin (**f**) and EMT markers including cytokeratin-7 (**g**) and vimentin (**h**)

**Fig. 5:**
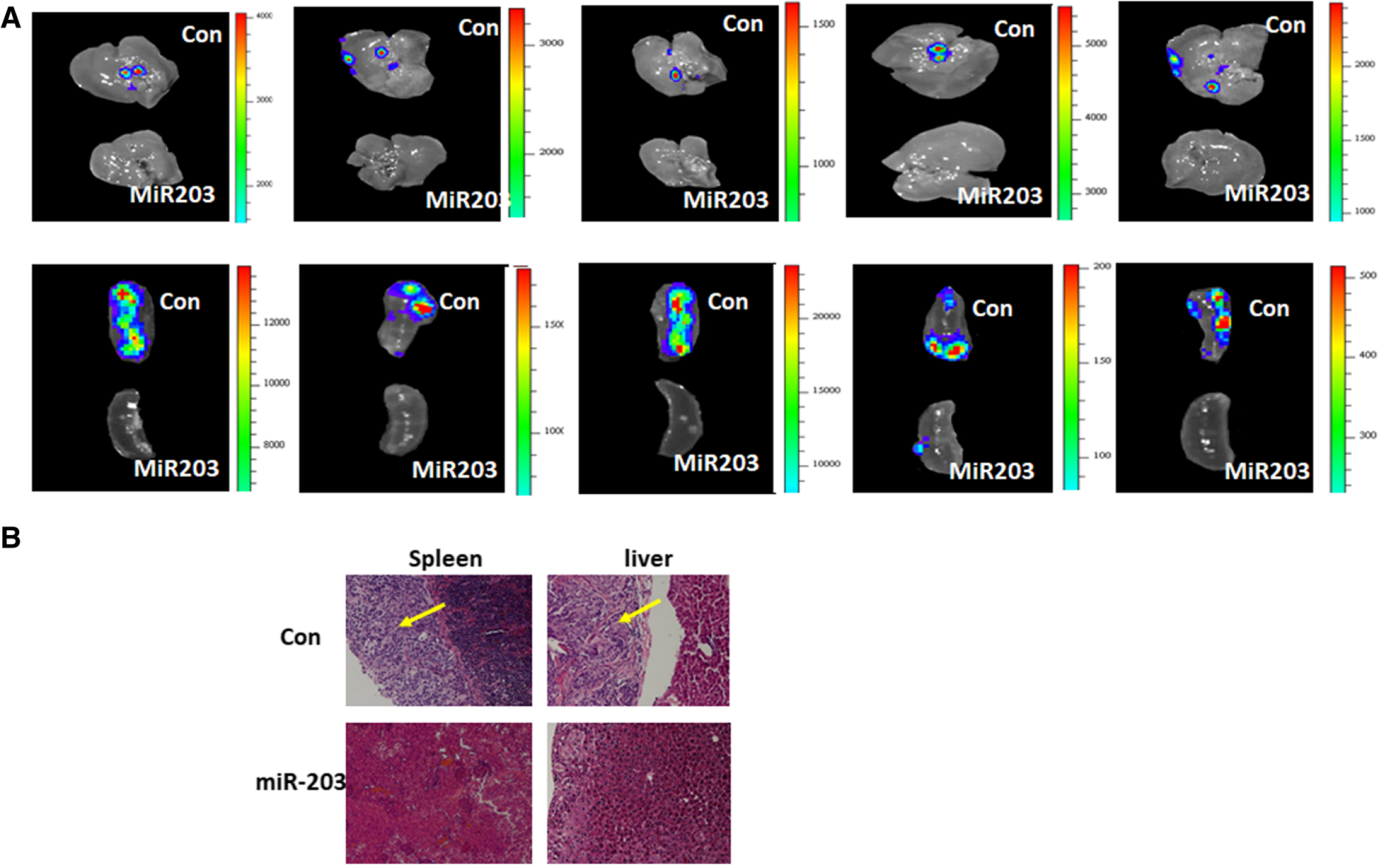
miR-203 expression inhibits ovarian tumor metastasis. **a**. Metastatic tumors in liver and spleen of NSG mice intrabursally injected with miR-203-expressing and control SKOV3 cells. **b**. H&E stained sections of tumor in liver and spleen of NSG mice intrabursally injected with miR-203-expressing and control SKOV3 cells

Although we showed previously that miR-203 expression inhibits ovarian tumor growth, it is not clear whether miR-203 expression affects ovarian tumor metastasis. Since miR-203 expression inhibits EMT, we hypothesized that miR-203 expression suppresses ovarian tumor metastasis. To test this hypothesis, we intrabursally injected miR-203-expressing and control SKOV3 cells labelled with luciferase into immunocompromised NSG female mice and examined primary tumor growth in ovaries and metastatic tumors in distant organs. Tumor growth in ovaries and dissemination into metastatic organs were monitored using live animal imaging after injecting D-luciferin at one month following cell injection (Fig. 4a). Mice intrabursally xenografted with miR-203-expressing SKOV3 cells showed significantly reduced tumor growth in ovaries (Figs. 4b and c). Tumors in ovaries were verified in H&E stained sections (Fig. 4d). Survivin and EMT markers were verified using Western blot from primary tumors and their expression levels were consistent with the data we obtained using cell lines. Survivin, mesenchymal markers, and phospho-SMAD2 were significantly reduced, whereas epithelial marker cytokeratin-7 was upregulated in tumors with miR-203 expression as compared to controls (Fig. 4e). In addition, we also performed immunostaining on primary ovary tumor sections using survivin, vimentin, and cytokeratin-7. Survivin and vimentin were weakly stained, whereas cytokeratin-7 was strongly stained in tumors with miR-203 expression compared to controls (Fig. 4f, g and h). We also examined tumor metastasis in these mice, and tumors were observed in multiple peritoneal organs including liver and spleen in control mice, but tumors were not detected in mice injected with miR-203-expressing SKOV3 cells (Fig. 5a). Invasion of tumors into liver and spleen were observed in H&E stained sections (Fig. 5b). These results demonstrate that miR-203 expression suppresses primary ovary tumor growth and metastasis by inhibiting EMT through attenuating the TGFβ pathway.

## Discussion

miRNAs function as tumor suppressors or oncogenes and are associated with tumor metastasis. In this study, we showed that miR-203 inhibited ovarian primary tumor growth and tumor metastasis by targeting survivin and attenuating the TGFβ pathway in an orthotopic ovarian cancer mouse model. Our findings validated the role of miR-203 in ovarian cancer by inhibiting primary ovarian tumor growth as a tumor suppressor and provided new experimental evidence that miR-203 inhibits ovarian tumor metastasis by suppressing EMT. miRNAs target multiple genes and downstream pathways. We previously showed that miR-203 inhibits tumor growth by directly targeting transcription factor Snai2 [23]. Here we demonstrated that miR-203-targeted BIRC5, whose expression is significantly upregulated in ovarian cancer compared to normal controls [24]. Therefore, miR-203, as a tumor-suppressing miRNA, inhibits EMT by targeting multiple genes and downstream pathways, thus suppressing tumor metastasis. Therefore, inducing miR-203 expression has therapeutic potential in the treatment of ovarian cancer.

miRNAs either function as tumor suppressors or oncogenes, depending on cellular context. miR-203 has been widely documented as functioning as a tumor-suppressing miRNA by targeting multiple oncogenes in different types of cancers including oral [28, 29], lung [30, 31], glioma [32], bladder [33, 34], gastric [35, 36], and colorectal cancers [37], ovarian [23], and breast cancers [38–41]. However, a few studies have found that miR-203 plays an oncogenic role in breast cancer [42, 43] or ovarian cancer [44].

Our results show that miR-203 inhibits ovarian cell migration and invasion. As a target gene of miR-203, survivin is highly expressed in multiple cancers including ovarian cancer, and the small molecule inhibitor of survivin YM155 has been in clinical trials for several cancer therapies [45–47]. We have previously shown that YM155 inhibited ovarian cell growth, migration and invasion [24]. In this study, we further show that miR-203 expression enhances the efficacy of YM155 in inhibiting BIRC5 expression, and also functionally in suppressing cell migration and invasion (Fig. 2). This result demonstrates that miR-203 can be used as an adjuvant for other chemotherapy drugs, including survivin inhibitors, to improve clinical therapy.

We demonstrated here that miR-203 expression inhibits ovarian tumor metastasis by suppressing EMT through targeting BIRC5, in addition through targeting Snai2, as we reported previously [23]. Recently, we have shown that BIRC5 expression promoted EMT in ovarian cancer cells [24]. Although it is not known how BIRC5 contributes to ovarian tumor metastasis, it is interesting to note that BIRC5 expression was downregulated in ovarian cancer cells by miR-203, suggesting that the miR-203/BIRC5 axis regulates ovarian tumor metastasis by inhibiting EMT.

We showed in this study that mice intrabursally injected with miR-203-expressing SKOV3 cells displayed significantly reduced primary tumor growth and tumor metastasis (Figs. 4 and 5), which validated our hypothesis that miR-203 expression indeed inhibits tumor metastasis by suppressing EMT. We showed previously that TGFβ promotes EMT in ovarian cancer cells [27]. For the first time, we demonstrated that miR-203 inhibited tumor metastasis by downregulating BIRC5 and attenuating the TGFβ pathway, BIRC5 is a direct target of miR-203. However, it is not clear how BIRC5 participates in the TGFβ pathway, which requires further investigation. XIAP (X-linked inhibitor of apoptosis protein), a member of the IAP family, directly interacts with TGFβ receptor1 (TGFβR1) through the BIR domain [48]. It is possible that survivin may activate the TGFβ pathway through interaction with the BIR domain of survivin in ovarian cancer cells.

## Conclusion

We provided new experimental evidence that miR-203 expression inhibits primary tumor growth in ovaries and peritoneal metastasis in an orthotopic ovarian cancer mouse model by attenuating the TGFβ pathway, thereby suppressing EMT.

## Additional file

Additional file 1: miR-203 enhances the efficacy of YM155 in the inhibion of ovarian cancer cell migration. (DOCX 940 kb)

## Abbreviations

BIRC5: Baculoviral IAP Repeat Containing 5
EMT: epithelial to mesenchymal transition
HCC: hepatocellular carcinoma
NSG: NOD *scid* gamma
