# Supplementary material for: miR-203 inhibits ovarian tumor metastasis by targeting BIRC5 and attenuating the TGFβ pathway
Source: J Exp Clin Cancer Res. 2018 Sep 21;37:235. doi: 10.1186/s13046-018-0906-0 (PMC6150978; doi:10.1186/s13046-018-0906-0)
Supplement: Supplementary file 1 — miR-203 enhances the efficacy of YM155 in the inhibion of ovarian cancer cell migration. (DOCX 940 kb) [file 13046_2018_906_MOESM1_ESM.docx]

**miR-203 inhibits ovarian tumor metastasis by targeting BIRC5 and attenuating the TGFβ pathway**

*Baojin Wang^1,2,3^, Xia Li^1,2,3^, Guannan Zhao^2,3^, Huan Yan^1,2,3^, Peixin Dong^6^, Hidemichi Watari^6^, Michelle Sims^2,3^, Wei Li^7^, Lawrence M. Pfeffer^2,3^, *Yuqi Guo^4,5^, *Junming Yue^2,3^

Correspondence to Junming Yue, Email: jyue@uthsc.edu


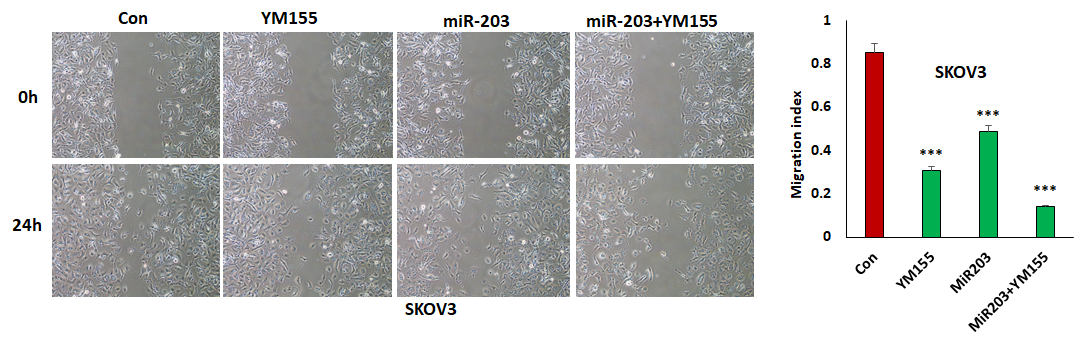
Fig.S1A. miR-203 expression significantly enhanced the efficacy of YM155 in inhibiting SKOV3 cell migration


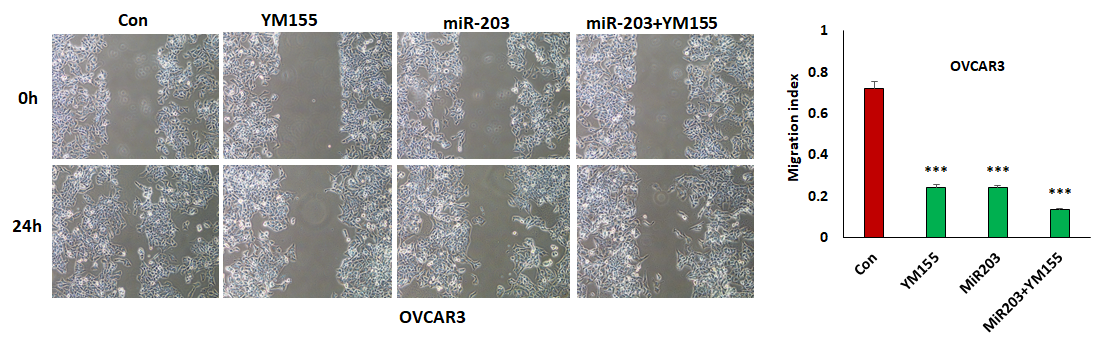
Fig.S1B. miR-203 expression significantly enhanced the efficacy of YM155 in inhibiting OVCAR3 cell migration
